# Real-World Data on the Effectiveness of Immunotherapy on Advanced NSCLC: A Retrospective Cohort Study

**DOI:** 10.3390/cancers18081239

**Published:** 2026-04-14

**Authors:** Antonios Katsarolis, Iliana Tapazidou-Spanoudi, Dimitris Kugiumtzis, Nikoleta Pastelli, Dionisios Spyratos, Katerina Manika, Anastasios Vagionas, Sofia Lampaki, Elena Fountzilas

**Affiliations:** 1 School of Medicine, Aristotle University of Thessaloniki, 54124 Thessaloniki, Greece; akatsarol@auth.gr; 2 Department of Biology, University of Rome Tor Vergata, 00133 Rome, Italy; iliana.tapazidouspanoudi@students.uniroma2.eu; 3 School of Electrical and Computer Engineering, Aristotle University of Thessaloniki, 54124 Thessaloniki, Greece; dkugiu@auth.gr; 4 Department of Pathology, “George Papanikolaou” General Hospital, 57010 Thessaloniki, Greece; pathologylab@gpapanikolaou.gr; 5 Oncology Unit, Pulmonology Clinic, “George Papanikolaou” General Hospital, School of Medicine, Aristotle University of Thessaloniki, 54124 Thessaloniki, Greece; dspyrato@auth.gr (D.S.); katman@auth.gr (K.M.); sofialampaki@auth.gr (S.L.); 6 Oncology Department, General Hospital of Kavala, 65500 Kavala, Greece; avagionas@auth.gr; 7 Department of Medical Oncology, St Luke’s Clinic, 55236 Thessaloniki, Greece

**Keywords:** non-small-cell lung cancer, immunotherapy, survival, real-world data, effectiveness

## Abstract

**Background/Objectives:** The aim of the study was to evaluate the real-world effectiveness of immunotherapy compared to chemotherapy in advanced non-small-cell lung cancer (NSCLC) and assess molecular profiling patterns in a large Greek cohort. **Methods:** This was a retrospective study of patients with advanced NSCLC from three oncology centers. Clinical, pathological, and/or molecular data were collected from the patient medical records. The primary endpoint was overall survival (OS). **Results:** Overall, 684 patients with advanced NSCLC were included; median age 67 years (range, 33 to 89). More than half of the patients (406, 59.4%) had been diagnosed with de novo metastatic disease. Overall, 289 of 684 (42.3%) patients underwent tumor molecular profiling. Immunotherapy use, with or without chemotherapy, in the first-line setting increased significantly over time (*p* < 0.001). Among 610 patients eligible for outcome analysis, immunotherapy at any line of treatment was associated with increased OS compared to chemotherapy alone (17.5 vs. 8.6 months; HR: 0.51, 95% CI: 0.42, 0.61; *p* < 0.001). The results remained consistent with the primary analysis as well as the landmark analysis using a 3-month cutoff to account for the immortal-time bias. Furthermore, time to next treatment (TTNT) was significantly longer with immunotherapy use in both first- and second-line treatment (TTNT1: 10.0 vs. 6.8 months, HR: 0.45, 95% CI: 0.34, 0.58; *p* < 0.001; TTNT2: 6.7 vs. 5.9 months, HR: 0.59, 95% CI: 0.40, 0.87; *p* = 0.009). Immunotherapy use remained an independent predictor of improved survival (HR: 0.50, 95% CI: 0.40, 0.63; *p* < 0.001). **Conclusions:** Immunotherapy, with or without chemotherapy, significantly improved clinical outcomes compared to chemotherapy alone in a real-world cohort of patients with advanced NSCLC. While molecular testing rates increased significantly over the study, only a minority of patients underwent PD-L1 testing, while broad molecular profiling was also incomplete, limiting the interpretation of treatment effects. Improvements to guarantee the universal molecular testing of patients with NSCLC are warranted.

## Simple Summary

Immunotherapy is now frequently used as a treatment for patients with advanced non-small-cell lung cancer. Questions arise in everyday clinical practice regarding the effectiveness of immunotherapy as monotherapy compared with its combination with chemotherapy, especially in subgroups of patients (i.e., older patients). Real-world data are particularly important to understand treatment outcomes in broader patient populations. The aim of this retrospective study was to assess the real-world impact of immunotherapy compared with chemotherapy in patients with advanced non-small-cell lung cancer, treated in Greece. The clinical, molecular, treatment and outcome data were collected from patients managed in routine oncology settings. This study demonstrated that immunotherapy at any line of treatment was associated with longer overall survival and longer time to next treatment compared with chemotherapy alone. The findings support the use of immunotherapy in real-world practice and highlight the importance of molecular profiling for optimizing patient care.

## 1. Introduction

Lung cancer remains the leading cause of cancer-related deaths worldwide, accounting for approximately 1.8 million deaths in 2020 [1]. In the United States alone, recent estimates indicate that around 230,000 people are diagnosed with lung cancer each year, while approximately 135,000 succumb to the disease [2]. Lung cancer continues to be the primary cause of cancer deaths in both male and female patients, surpassing the combined fatalities from prostate, breast, brain, and colorectal cancers [1].

Although lung cancer remains a significant health challenge, mortality rates have been declining due to smoking cessation efforts, expanded screening programs, and advancements in medical oncology [3]. Notably, new therapeutic approaches, particularly immunotherapy, have revolutionized treatment for advanced non-small-cell lung cancer (NSCLC). Immunotherapy has significantly improved survival rates, offering prolonged life expectancy and improved quality of life for selected patients. Nevertheless, real-world studies evaluating the effectiveness of immunotherapy for various patient groups, while also providing prognostic and/or predictive biomarkers, are limited [4,5]. Furthermore, the evidence on the use of molecular analysis methods to guide treatment in real-world settings remains insufficient [6].

Real-world data studies provide clinically relevant information by evaluating treatment options in unselected and heterogenous patient populations that better reflect everyday practice. Unlike randomized clinical trials, they capture outcomes in patients with comorbidities, poor performance status, and other subgroups often excluded from clinical trials, while further reinforcing clinical trial data. Therefore, real-world evidence is crucial in understanding the true effectiveness of immunotherapy in advanced NSCLC [4].

This study aimed to assess the real-world effectiveness of immunotherapy in a large cohort of patients with advanced NSCLC, while providing valuable insights into its impact on disease outcomes. It also highlighted the impact of molecular profiling in decision-making and treatment personalization, emphasizing the need for universal testing among patients with advanced NSCLC.

## 2. Materials and Methods

### 2.1. Population

This was a retrospective observational study across three oncology centers affiliated with the Hellenic Cooperative Oncology Group (HeCOG). The study included patients diagnosed with advanced NSCLC at any point in their disease course. Patients could have recurred after being diagnosed with early-stage disease or advanced disease (stage IIIB, IIIC or IV). Patients with any NSCLC histological subtype were included. Waiver of consent was obtained from the Institutional Review Board of “G. Papanikolaou” General Hospital for the use of pseudoanonymized patient data (protocol number: 419/2.6.2022).

### 2.2. Data Collection

**Figure 1:**
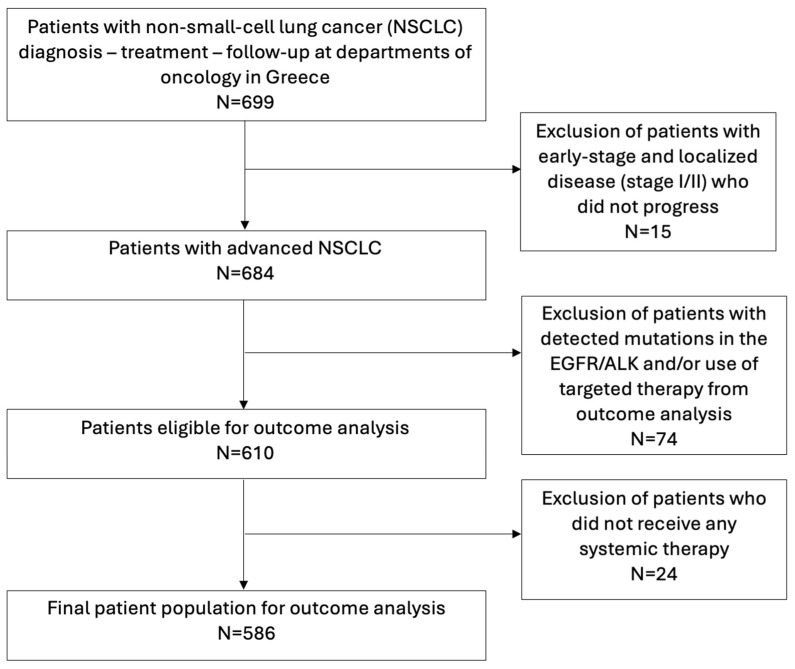
Consort diagram.

Data from the patients’ medical records maintained at each participating center were retrospectively collected. These included demographics; clinical, pathological and molecular characteristics; along with treatment and outcome data. No data on adverse events were recorded. Patients initially diagnosed with early-stage NSCLC, where disease progression was not established, were excluded from the analysis. Tumor molecular profiling data refer to all patients in the cohort with advanced disease, whereas patients harboring EGFR or ALK mutations and/or receiving standard targeted therapy were excluded from outcome analyses, while a small number receiving other targeted agents were retained (Figure 1, Supplementary Table S1). Patients were also excluded from the analysis if they had not received any systemic treatment. Treatment decisions were based on clinical judgment, patient characteristics, and treatment availability during the respective time periods. The reporting of this study followed the ESMO GROW recommendations for the presentation and interpretation of real-world evidence [7].

### 2.3. Statistical Analysis

The primary endpoint of the study was overall survival (OS) between patients with advanced NSCLC who received immunotherapy alone or in combination with chemotherapy, compared to those who did not receive immunotherapy at any line of treatment. In this analysis patients not receiving any systemic treatment were not included in the analysis. OS was defined as time from diagnosis of advanced disease to death or last contact. In order to mitigate the impact of immortal-time bias we introduced a separate landmark analysis, using a predefined cutoff of three months. Secondary endpoints included time to next treatment (TTNT), evaluation of molecular profiling practices over time and exploration of predictive factors for immunotherapy. TTNT1 and TTNT2 were defined as the time interval from the initiation of first- or second-line treatment, respectively, to the date of initiation of next treatment, death from any cause or last contact, whichever occurred first. Patients harboring EGFR or ALK mutations were excluded from the final outcome analysis regarding immunotherapy benefit.

Survival curves were generated using the Kaplan–Meier method. Univariate and multivariate analyses of independent prognostic factors were performed using the Cox proportional hazards model (Cox, 1972 [8]). Multivariable models included age, sex, histology, performance status, smoking status, stage, and calendar period to account for temporal bias. Specifically, calendar period was included as a categorical variable with two major time groups (1999–2018, 2019–2024). Association of a variable of interest, e.g., immunotherapy as a first-line option, with time periods was analyzed using the Pearson chi-squared test. Statistical analysis was conducted using Matlab software (MathWorks, Inc. MATLAB R2023a, Natick, MA, USA).

## 3. Results

### 3.1. Patient Characteristics

Overall, 699 patients diagnosed with NSCLC between November 1999 and November 2024 were included in the study. The majority were men (581 patients, 83.1%). The median age was 67 years (range, 33 to 89). Of these, 684 patients (97.9%) were diagnosed with advanced disease (stage III or IV) at some point during their clinical course, while 57.2% (391 of 684) had been diagnosed with de novo metastatic disease. The majority of patients were former or current smokers (607, 89.1%). Adenocarcinoma was the most common histologic subtype (375 patients, 53.7%), followed by squamous cell carcinoma (277 patients, 39.6%). A detailed overview of patient characteristics is provided in Table 1.

### 3.2. Tumor Molecular Profiling

A total of 289 of 684 patients (42.3%) underwent tumor molecular profiling during the course of their disease. The number of genes tested in each patient was not determined in the majority of patients. The rate of testing (any gene) increased significantly over time: no patients underwent tumor molecular testing between 1999 and 2008, followed by 17 patients (2.5%) who underwent any testing between 2009 and 2013, 90 patients (13.2%) between 2014 and 2018, and 182 patients (26.6%) between 2019 and 2024 (*p* < 0.001). Histological subtype was significantly associated with the performance of molecular testing (*p* < 0.001), with patients with adenocarcinoma undergoing molecular testing more frequently compared with squamous cell carcinoma and other histological subtypes. Adenocarcinomas accounted for 77.2% of all molecularly tested cases, whereas squamous cell carcinomas and other histological subtypes represented 17.6% and 5.2%, respectively. Pathogenic driver mutations were identified in 111 patients (38.4%). Among those with actionable or pathogenic mutations, the most commonly affected genes were EGFR (18.3%) and KRAS (9.7%), followed by HER2 (1.4%), ALK (1.0%) and BRAF (0.7%). Notably, these numbers represent positive results among the patients who were tested for any gene. PD-L1 expression was evaluated in 245 patients (35.8%). Among these, PD-L1 expression was ≥50% in 19.6%, 1–50% in 30.2%, and <1% in 50.2% of patients. A summary of molecular testing results is shown in Table 2.

### 3.3. Treatment Regimens

**Figure 2:**
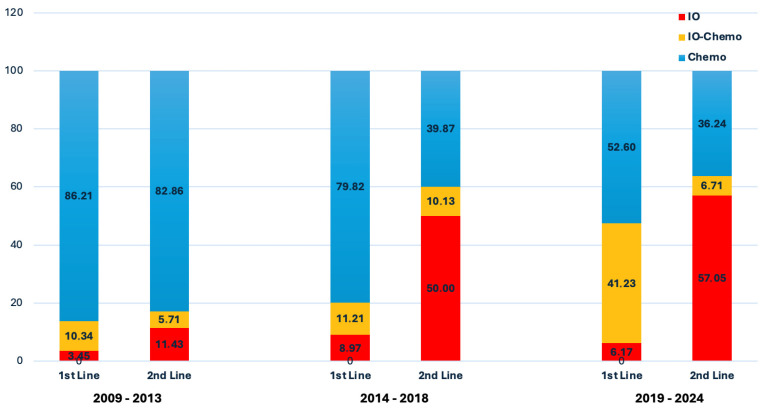
Distribution of treatment regimens over time. IO: immunotherapy monotherapy; IO–Chemo: immunotherapy–chemotherapy combination; Chemo: chemotherapy.

Among the 660 patients who received treatment, 400 (60.6%) received chemotherapy as first-line treatment, 159 (24.1%) received a combination of chemotherapy and immunotherapy, 41 (6.2%) received immunotherapy alone, and 60 (9.1%) received targeted therapies. Targeted agents included mainly afatinib (N = 34 patients) followed by osimertinib (N = 22) and erlotinib (N = 11). A detailed view of the targeted therapy regimens chosen at any line is presented in Supplementary Table S1. A subset of patients (24, 3.5%) succumbed to the disease prior to the initiation of any systemic treatment. In total, 363 (53.1%) patients received second-line therapy, consisting of immunotherapy as monotherapy in half of the cases (172 of 363, 47.4%). In addition, 48 (7.3%) patients received immunotherapy in more than one line of treatment. The distribution of treatment regimens across lines of treatment is depicted in Supplementary Table S2. Overall, 409 (62.0%) patients received immunotherapy at some point during their treatment. Overall, the use of immunotherapy showed a steady increase throughout the study period. In particular, first-line immunotherapy use significantly increased over time (0 of 4 patients in 1999–2003, 1 of 7 in 2004–2008, 8 of 60 in 2009–2013, 45 of 253 in 2014–2018, and 146 of 336 in 2019–2024; *p* < 0.001) (Figure 2, Supplementary Figure S1).

### 3.4. Clinical Outcomes

**Figure 3:**
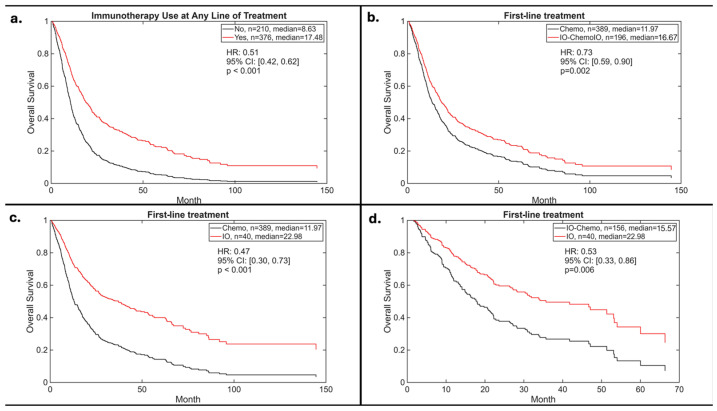
Overall survival: (**a**) immunotherapy at any treatment line vs. no immunotherapy; (**b**) first-line chemotherapy alone vs. immunotherapy (alone or combined with chemotherapy); (**c**) first-line chemotherapy alone vs. immunotherapy alone; (**d**) first-line combination immunotherapy plus chemotherapy vs. immunotherapy alone.

At a median follow-up of 13.4 months, patients who received immunotherapy in any line of treatment, either as monotherapy or in combination, demonstrated a significantly prolonged median OS of 17.5 months compared with 8.6 months among patients who did not receive immunotherapy (hazard ratio [HR]: 0.51, 95% confidence interval [CI]: 0.42–0.62; *p* < 0.001) (Figure 3a).

In the landmark analysis using a 3-month cutoff, patients who survived beyond the landmark time were included in the analysis. The results remained consistent with the primary analysis, showing that patients receiving immunotherapy at any line showed improved OS compared to chemotherapy regimens (mOS 18.1 vs. 10.4 months; HR: 0.55, 95% CI: 0.45–0.68; *p* < 0.001). No substantial differences in effect estimates were observed, suggesting that immortal-time bias did not significantly influence the findings (Supplementary Figure S2).

In the first-line setting, immunotherapy-containing regimens (monotherapy or combined with chemotherapy) were associated with improved survival compared to chemotherapy alone (mOS 16.7 vs. 12.0 months; HR: 0.73, 95% CI: 0.60–0.90; *p* = 0.002) (Figure 3b).

Notably, first-line immunotherapy as monotherapy was associated with the greatest survival benefit. Compared to chemotherapy alone, immunotherapy as monotherapy significantly reduced the risk of death (HR: 0.47, 95% CI: 0.30–0.73; *p* < 0.001), with a median OS of 23 months versus 12 months (Figure 3c). Furthermore, immunotherapy monotherapy was also superior to combination chemotherapy/immunotherapy, demonstrating improved survival (median OS 23 vs. 15.6 months; HR: 0.53, 95% CI: 0.33–0.86; *p* = 0.005) (Figure 3d).

The line of immunotherapy administration did not significantly impact OS. Patients receiving immunotherapy in the first-line setting had a median OS comparable to those receiving immunotherapy in later lines (16.8 vs. 17.4 months; HR: 1.07, 95% CI 0.83–1.38; *p* = 0.591).

**Figure 4:**
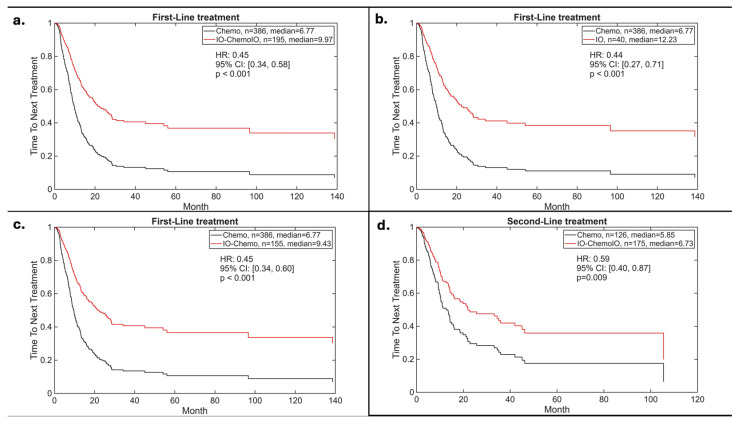
Time to next treatment (TTNT): (**a**) first-line immunotherapy (alone or combined with chemotherapy) vs. chemotherapy alone; (**b**) first-line immunotherapy alone vs. chemotherapy alone; (**c**) first-line combination immunotherapy plus chemotherapy vs. chemotherapy alone; (**d**) second-line immunotherapy (alone or combined with chemotherapy) vs. chemotherapy alone.

Similarly, median TTNT in the first-line setting was longer in patients receiving immunotherapy vs. chemotherapy (10.0 vs. 6.8 months, HR: 0.45, 95% CI 0.34–0.58; *p* < 0.001) (Figure 4a). Specifically, when compared to chemotherapy, either immunotherapy alone (mTTNT 12.2 months versus 6.8 months, HR: 0.44, 95% CI 0.27–0.71; *p* < 0.001) (Figure 4b) or in combination with chemotherapy (mTTNT 9.4 months versus 6.8 months, HR: 0.45, 95% CI 0.34–0.60; *p* < 0.001) (Figure 4c) was associated with a longer duration before treatment change. Median TTNT was also longer in patients receiving immunotherapy (6.7 months) as second-line therapy vs. chemotherapy patients (5.9 months) (HR: 0.59, 95% CI 0.40–0.87; *p* = 0.009) (Figure 4d). TTNT outcomes were consistent across PD-L1 expression subgroups, with numerically longer TTNT observed in patients with PD-L1 ≥50%.

A multivariate Cox proportional hazards regression analysis was performed to identify independent predictors of OS. Receipt of immunotherapy at any line of treatment remained independently associated with improved OS (HR: 0.46, 95% CI: 0.36–0.58; *p* < 0.001) (Table 3a). Immunotherapy use at any line showed independent improvement in OS when accounting for landmark analysis (HR: 0.53, 95% CI: 0.41–0.69, *p* < 0.001) (Table 3b). Similarly, receipt of immunotherapy in the first-line treatment was independently associated with improved OS (HR: 0.68, 95% CI: 0.53–0.87; *p* = 0.002) (Table 3c). A separate multivariate analysis including PD-L1 status confirmed the independent association of immunotherapy with improved OS, with PD-L1 positivity also emerging as a significant predictor (Supplementary Table S3).

## 4. Discussion

The introduction of immune checkpoint inhibitors targeting the PD-1/PD-L1 axis has fundamentally ameliorated the prognosis of patients with advanced disease, offering not only a substantially improved quality of life but also longer survival. To our knowledge, this is the largest retrospective real-world study for the use of immunotherapy on Greek patients with NSCLC.

In our cohort, the administration of immunotherapy at any line of treatment was associated with longer OS and TTNT, supporting its clinical benefit across real-world settings. Interestingly, immunotherapy monotherapy appeared to have higher OS compared to chemo-immunotherapy combinations. This observation should be interpreted cautiously given the retrospective design and potential confounding by indication and patient selection. Patients receiving combination regimens often had lower PD-L1 expression or more aggressive disease, with a higher disease burden, while immunotherapy monotherapy was predominantly administered as a first-line treatment in patients with PD-L1 ≥50%, reflecting current clinical practice guidelines and potentially explaining the observed differences in outcome. However, it underscores the importance of optimal patient selection and biomarker-driven treatment decisions.

In our study, TTNT was used as a pragmatic real-world surrogate for progression-free survival [9], with the understanding that it can be influenced not only by disease progression but also by physician practice, patient preference, treatment access, and toxicity. Despite these limitations, TTNT was significantly longer in patients receiving immunotherapy in both first- and second-line settings, with numerically superior outcomes when immunotherapy was administered earlier in the disease course. These findings suggest that early exposure to immunotherapy may translate into more durable disease control, consistent with the concept of front-loading effective systemic therapy [10].

Importantly, OS was significantly improved in patients who received immunotherapy at any point during their treatment course compared to the rest of the patients. Patients exposed to immunotherapy demonstrated a doubling of median OS compared with those who never received immunotherapy (17.5 vs. 8.6 months), an effect that remained robust in multivariate analysis after accounting for age, sex, histology, stage, performance status, smoking status and calendar period. This survival advantage associated with immunotherapy was also evident when administered in the first-line setting. These results are consistent with previously published real-world and clinical trial data, confirming that the survival benefit of immune checkpoint inhibitors extends beyond selected trial populations into routine clinical practice. Specifically, first-line immunotherapy, whether as monotherapy [10,11] or in combination with chemotherapy [12,13,14,15], has been demonstrated to be associated with improved outcomes. Several trials with previously treated patients with advanced NSCLC, including CheckMate-017, 057, KEYNOTE-010 and OAK, have also demonstrated a clear benefit from immunotherapy compared to chemotherapy [16,17,18,19]. These findings reinforce the central role of immunotherapy as a backbone in the management of advanced NSCLC, regardless of line.

In our cohort, there were no statistically significant differences observed in OS between patients receiving immunotherapy, either as monotherapy or as a combination with chemotherapy regimens, as first-line treatment versus later lines. Several factors may explain this finding. First of all, the widespread use of immunotherapy for the treatment of the disease across all lines of treatment is considered a routine practice and may have resulted in a “cross-over” effect. This means that many patients not treated with immunotherapy upfront ultimately received it later, attenuating differences in survival [20]. Moreover, patients who are fit to receive second-line therapy may represent a more favorable subgroup of patients with a better performance status and disease biology, therefore counterbalancing the advantages of an earlier introduction of immunotherapy on their treatment. Conversely, patients with rapidly progressing disease often fail to receive second or subsequent lines of treatment. This was also observed in our cohort, in which a substantial proportion of patients never received second-line therapy, resulting in an enrichment of better responders among those who did not receive first-line immunotherapy [21]. Taken together, these findings emphasize the importance of administering immunotherapy upfront. Finally, the heterogeneity of treatment patterns in real-world settings, either regarding monotherapy or combination therapies across multiple lines of treatment, may have further confounded potential outcome differences [22,23,24].

Subgroup analysis by age, using 70 years as a cutoff, revealed that although older patients may experience numerically smaller benefits—potentially due to comorbidities, altered tumor biology, or immunosenescence—immunotherapy remained beneficial compared to chemotherapy. No statistically significant differences in OS or TTNT were observed between older and younger patients, corroborated by other published studies [10,18,25]. Specifically, pooled analyses of older patients in KEYNOTE-010/024/042 demonstrated comparable efficacy and manageable safety in PD-L1-positive NSCLC, while consensus guidance supports that chronological age alone should not preclude immunotherapy treatment for advanced NSCLC.

Our findings are consistent with a growing body of real-world evidence supporting the effectiveness of immunotherapy across all treatment lines in advanced NSCLC. The French IFCT-1502 CLINIVO study of second- or later-line nivolumab in a large unselected population of patients with refractory NSCLC reported a median OS of 9.7 months, confirming a robust benefit beyond the clinical trial setting and in a more challenging group of patients [26]. Similarly, Hektoen et al. demonstrated a survival benefit of pembrolizumab on a Norwegian population database over conventional chemotherapy regimens similar to that of the clinical trials used for the approval of the drug [27]. Furthermore, propensity score-matched analyses of over 10,000 patients demonstrated that immunotherapy significantly improved OS and delayed TTNT compared with chemotherapy, both in first- and second-line settings [28]. Finally, similar registry-based studies in Europe further reinforce the prolonged survival of immunotherapy regimens across diverse population groups, while highlighting a particularly increased benefit in those with a better performance status and consistent real-world toxicities with those of clinical trials [29,30]. Collectively, these data validate that immunotherapy can be administered in a broader and more complex group of patients encountered in routine clinical practice offering a prolonged survival and a better quality of life.

Lastly, molecular profiling plays a pivotal role in precision oncology, enabling the selection of the optimal treatment in each patient [31]. In Greece, routine reimbursement of three biomarkers (KRAS, EGFR, ALK) in patients with NSCLC was initiated in 2019. Therefore, to date only a proportion of patients with NSCLC undergo molecular testing with broad NGS panels, despite full reimbursement of all approved EMA therapeutic regimens [32]. This gap between approved treatments and access to molecular testing limits the implementation of precision medicine in NSCLC. Indeed, in the outpatient population, a significant increase in molecular testing was observed over time. In October 2025, the Greek Government enacted a new ministerial decision published in the Government Gazette (“ΦΕΚ Β’ 5627”) expanding biomarker reimbursement and facilitating broader access to molecular diagnostics for patients with advanced NSCLC, thereby ensuring that a greater number of patients will have access to guideline-recommended testing at diagnosis and across treatment lines. This regulatory change is expected to reduce barriers to precision oncology and align real-world practice with contemporary standards of care [33].

This study has several limitations, the most important being its retrospective design. First, the design and the data collection introduce inherent selection bias and temporal confounding, especially given the long study period (1999–2024). Differences in treatment availability over time; incomplete molecular profiling, especially during the early years; and the absence of systematic adverse event data further limit generalizability of the findings. Another important confounding factor was the small subset of patients undergoing PD-L1 testing, which in combination with the diverse molecular testing may have influenced treatment selection and outcome interpretation. TTNT outcomes must also be viewed cautiously as a surrogate for PFS due to the influence of this endpoint not only by disease progression. A further limitation of this study is the lack of available data on adverse events and treatment-related toxicities, which are particularly relevant in real-world settings. The absence of such information precludes a comprehensive assessment of the risk–benefit profile of the evaluated treatments and may limit the interpretation of their overall clinical utility. Finally, other important factors such as metastatic burden, oncology center and de novo versus recurrent disease could not be assessed, which may have influenced the outcome analysis.

## 5. Conclusions

This multicenter retrospective analysis represents the largest real-world study of immunotherapy in patients with NSCLC in Greece to date. Our findings validate that the administration of immunotherapy, whether as monotherapy or in combination with chemotherapy, provides a significant OS benefit compared to chemotherapy alone, regardless of the line of treatment. This survival advantage persists in a heterogenous, unselected patient population, reinforcing the external validity of pivotal randomized clinical trials. Furthermore, the study identifies performance status and tumor stage as critical independent prognostic factors, whereas smoking history showed only a trend toward poorer survival without statistical significance in the multivariate model. However, these results need to be interpreted with caution due to the limited availability of PD-L1 testing and molecular profiling, which may have influenced treatment selection.

Additionally, our data highlight the evolving landscape of tumor molecular profiling and biomarker identification. While there has been a significant increase in molecular testing rates over the last decade, a discrepancy remains between the availability of approved targeted therapies and the implementation of broad molecular profiling. The recent regulatory expansion of biomarker reimbursement in Greece is expected to bridge this gap, facilitating optimal treatment selection.

## Figures and Tables

**Table 1:** Patient characteristics.

Characteristic		Number of Patients (%)
Age at Diagnosis N = 699	Median: 67 years	
	Range: 33–89 years	
Sex N = 699	Male	581 (83.1%)
	Female	118 (16.9%)
Smoking Status N = 681	Former or Current	607 (89.1%)
	Never	74 (10.9%)
Histology N = 699	Adenocarcinoma	375 (53.7%)
	Squamous	277 (39.6%)
	Other	47 (6.7%)
ECOG N = 546	0	371 (68.0%)
	1	148 (27.1%)
	≥2	27 (4.9%)
Stage At Initial Diagnosis N = 699	I	28 (4.0%)
	II	28 (4.0%)
	III	237 (33.9%)
	IV	406 (58.1%)

**Table 2:** Tumor molecular profiling data.

		Number of Patients (%)
Molecular profiling test	Yes	289 (42.3%)
	No	395 (57.8%)
Pathogenic mutation	Yes	111 (38.4%)
	No	178 (61.6%)
≥2 concurrent mutations	Yes	16 (14.4%)
	No	95 (85.6%)
Genes affected	KRAS	28
	EGFR	53
	ALK	3
	HER-2	4
	BRAF	2
	RET	2
	MEK	2
	MET	2
	TP53	17
	PIK3CA	4
	RB1	2
	NRAS	2
	CTNNB1	1
	FGFR	1
	FGFR3	1
	HRAS	1
	BRCA2	1
	PMS2	1
	PTEN	1
	STK11	1
PD-L1 testing	Yes	245 (35.8%)
	No	439 (64.2%)
PD-L1 expression	Negative (<1%)	123 (50.2%)
	Positive (1–49%)	74 (30.2%)
	Highly positive (≥50%)	48 (19.6%)

**Table 3:** Multivariate Cox regression for immunotherapy use at any line of treatment.

Variables	HR	*p*-Value
(a) Multivariate Cox Regression for Immunotherapy Use at Any Line of Treatment		
IO at Any Line	0.46	<0.001
Smoking Status	1.67	0.026
Stage at Diagnosis	2.05	0.001
Performance Status	1.39	0.006
Age at Diagnosis	1.09	0.463
Gender	0.93	0.669
Histology	0.90	0.340
Calendar Period	1.41	0.004
(b) Multivariate Cox Regression for Immunotherapy Use at Any Line of Treatment (Landmark Analysis)		
IO at Any Line	0.53	<0.001
Smoking Status	1.64	0.045
Stage at Diagnosis	1.85	0.006
Performance Status	1.33	0.032
Age at Diagnosis	1.07	0.556
Gender	0.97	0.872
Histology	0.91	0.432
Calendar Period	1.29	0.048
(c) Multivariate Cox Regression for First-Line Treatment		
First-Line Treatment Scheme	0.68	0.002
Smoking Status	1.50	0.080
Stage at Diagnosis	1.64	0.025
Performance Status	1.39	0.008
Age at Diagnosis	1.05	0.659
Gender	0.96	0.803
Histology	0.91	0.413
Calendar Period	1.42	0.007

Abbreviations: HR: hazard ratio; IO: immunotherapy.

## Data Availability

Data will be available upon request.

## Supplementary Materials

The following supporting information can be downloaded at: https://www.mdpi.com/article/10.3390/cancers18081239/s1, Figure S1: Immunotherapy use trends over time. Figure S2: Immunotherapy use at any line of treatment. Table S1. Targeted Therapy Regimens. Table S2. (a) First-Line Treatment Regimens (b) Second-Line Treatment Regimens. Table S3. Multivariate Cox Regression including PD-L1 status.

## Abbreviations

The following abbreviations are used in this manuscript:

NSCLC	Non-small-cell lung cancer
OS	Overall survival
mOS	median overall survival
TTNT	Time to next treatment
mTTNT	Median time to next treatment
PFS	Progression-free survival
IO	Immunotherapy
IO-Chemo	Immunotherapy–Chemotherapy Combination
Chemo	Chemotherapy
HeCOG	Hellenic Cooperative Oncology Group
ECOG	Eastern Cooperative Oncology Group
