# Supplementary material for: Real-World Data on the Effectiveness of Immunotherapy on Advanced NSCLC: A Retrospective Cohort Study
Source: Cancers (Basel). 2026 Apr 14;18(8):1239. doi: 10.3390/cancers18081239 (PMC13115517; doi:10.3390/cancers18081239)
Supplement: Supplementary file 1 [file cancers-18-01239-s001.zip › Supplementary Material.pdf]

Supplementary Material: Real- World Data on the Efficacy of Immunotherapy on Advanced NSCLC: A retrospective cohort study

Supplementary Table S1. Targeted Therapy Regimens

Targeted Therapy Regimen	Number of Patients
Afatinib	34
Osimertinib	22
Erlotinib	11
Gefitinib	6
Crizotinib	3
Sotorasib	2*
Alectinib	1
Lapatinib	1*
Cabozatinib	1*
Nintedanib	1*
Nipararib	1*

***The patients receiving these targeted agents were not excluded from the outcome analysis**

Supplementary Table S2. (a). First-Line Treatment Regimens. **(b).** Second-Line Treatment Regimens.

(a)

Regimen Type	Number	Percentage (%)
Chemotherapy	400	60.6
Immunotherapy	41	6.2
Immunotherapy-Chemotherapy Combination	159	24.1
Targeted Agents	60	9.1

(b)

Regimen Type	Number	Percentage (%)
Chemotherapy	149	41.0
Immunotherapy	172	47.4
Immunotherapy-Chemotherapy Combination	28	7.7
Targeted Agents	13	3.6
Other	1	0.3

Supplementary Table S3. Multivariate Cox Regression including PD-L1 status.

Variables	HR	p- value
IO At Any Line	0.49	0.011
Smoking Status	1.80	0.105
Stage At Diagnosis	1.74	0.318
Performance Status	1.42	0.106
Age At Diagnosis	1.10	0.647
Gender	1.33	0.348
Histology	0.75	0.182
Calendar Period	1.57	0.129
PD-L1 Status	0.53	0.002
