# Supplementary figures and images for: Real-World Data on the Effectiveness of Immunotherapy on Advanced NSCLC: A Retrospective Cohort Study
Source: Cancers (Basel). 2026 Apr 14;18(8):1239. doi: 10.3390/cancers18081239 (PMC13115517; doi:10.3390/cancers18081239)

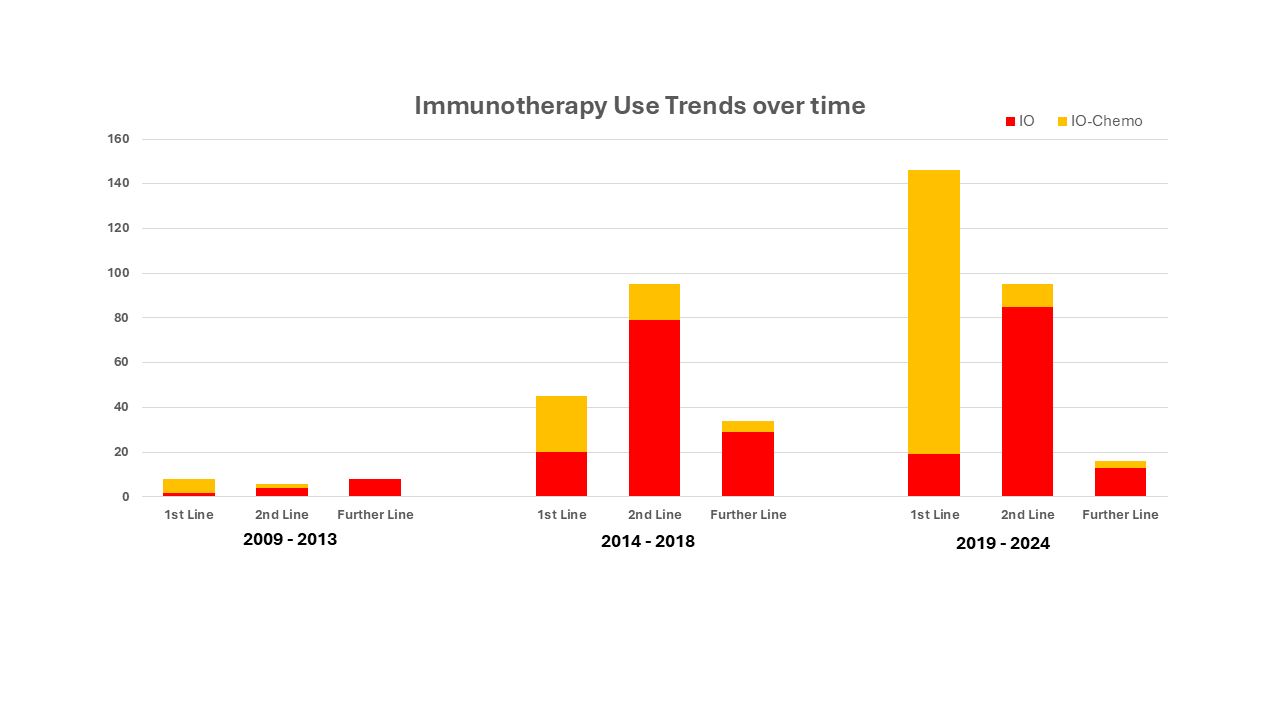

Supplement: Supplementary file 1 [file cancers-18-01239-s001.zip › Supplementary Figure S1.tif]

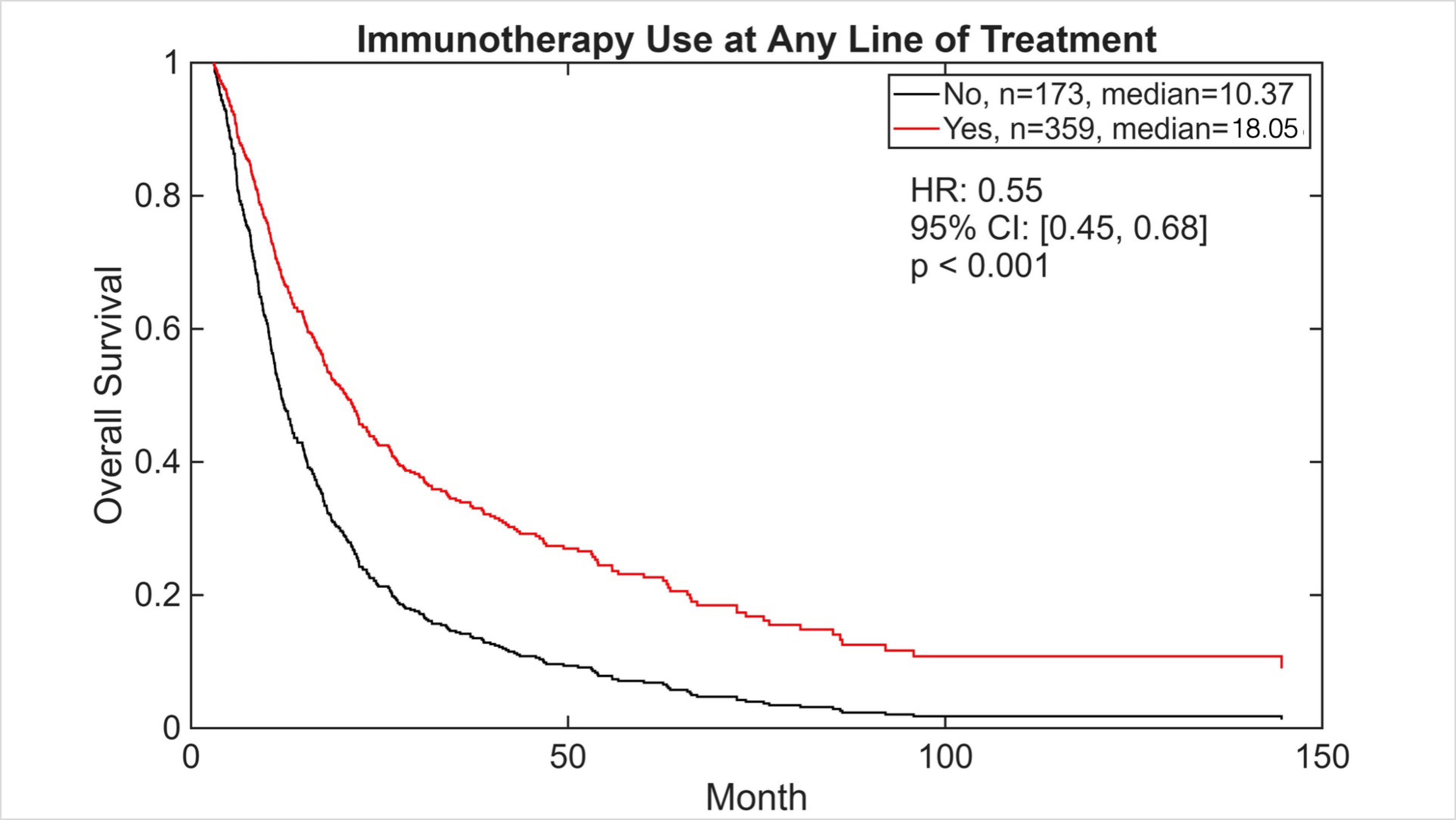

Supplement: Supplementary file 1 [file cancers-18-01239-s001.zip › Supplementary Figure S2.jpeg]
